# Risk factors for complications and mortality of percutaneous endoscopic gastrostomy insertion

**DOI:** 10.1186/s12876-018-0825-8

**Published:** 2018-06-28

**Authors:** Gyu Young Pih, Hee Kyong Na, Ji Yong Ahn, Kee Wook Jung, Do Hoon Kim, Jeong Hoon Lee, Kee Don Choi, Ho June Song, Gin Hyug Lee, Hwoon-Yong Jung

**Affiliations:** Department of Gastroenterology, Asan Medical Center, University of Ulsan College of Medicine, 88, Olympic-ro 43-gil, Songpa-gu, Seoul, 05505 Korea

**Keywords:** Stomach, Endoscopy, Gastrostomy, Complication, Mortality

## Abstract

**Background:**

Percutaneous endoscopic gastrostomy (PEG) is a relatively safe procedure; however, acute and chronic complications of PEG have been reported. We aimed to determine risk factors associated with complications and 30-day mortality after PEG, based on 11 years of experience at a single tertiary hospital.

**Methods:**

In total, 401 patients who underwent first PEG insertion at the Asan Medical Center, Seoul, Korea, between January 2005 and December 2015 were eligible. Medical records were retrospectively reviewed to determine clinical characteristics and outcomes of 139 and 262 patients who underwent pull-type and introducer-type PEG, respectively.

**Results:**

The median age of the overall population was 68 years, and the median body mass index was 19.5 kg/m^2^. Acute and chronic complications developed in 96 (23.9%) and 105 (26.2%) patients. Acute ileus and chronic tube obstruction were significantly more frequent in the introducer-type PEG group (*p* = 0.033 and 0.001, respectively). The 30-day mortality rate was 5.0% (median survival: 10.5 days). Multivariate analysis revealed that underlying malignancy was a predictor of acute complications; age ≥ 70 years and diabetes mellitus were predictors of chronic complications. The median follow-up was 354 days. Neurologic disease and malignancy were the most common indications for PEG. Neurologic diseases were classified into two groups: stroke and the other neurologic disease group (including dementia, Parkinson’s disease, neuromuscular disease, and hypoxic brain damage). Multivariate analysis showed that 30-day mortality was significantly lower in the other neurologic disease group and higher in patients with platelet count < 100,000/μL, and C-reactive protein (CRP) ≥ 5 mg/dL.

**Conclusions:**

PEG is a relatively safe and feasible procedure, but it was associated with significantly higher early mortality rate in patients with platelet count < 100,000/μL or CPR≥5mg/dL, and lower early mortality rate in neurologic disease group including dementia, Parkinson's disase, neuromuscular disease, and hypoxic brain damage. In addition, acute complications in patients with underlying malignancy, and chronic complications in patients aged ≥70 and those with diabetes mellitus should be considered during and after PEG.

## Background

Percutaneous endoscopic gastrostomy (PEG) is the most common method of enteral nutrition in patients who are expected to require supplementary enteral feeding for longer than 2–3 weeks [1]. Since its introduction in the 1980s, PEG has been associated with superior outcomes with respect to complication and mortality rates compared with radiological or surgical gastrostomy [2, 3]. Although PEG is a relatively safe and minimally invasive procedure, acute and chronic complications have been reported. In addition, the 30-day mortality after PEG has been reported to be 3.3–23.9% [4–9]. Therefore, the identification of risk factors associated with complications and early mortality is crucial when evaluating which patients will benefit most from PEG procedures. We aimed to identify potential risk factors associated with acute complications, chronic complications and 30-day mortality after PEG using data from a large number of patients who underwent tube insertion using the pull- or introducer-type PEG technique during an 11-year period at a single center in Korea.

## Methods

### Patients

**Fig. 1:**
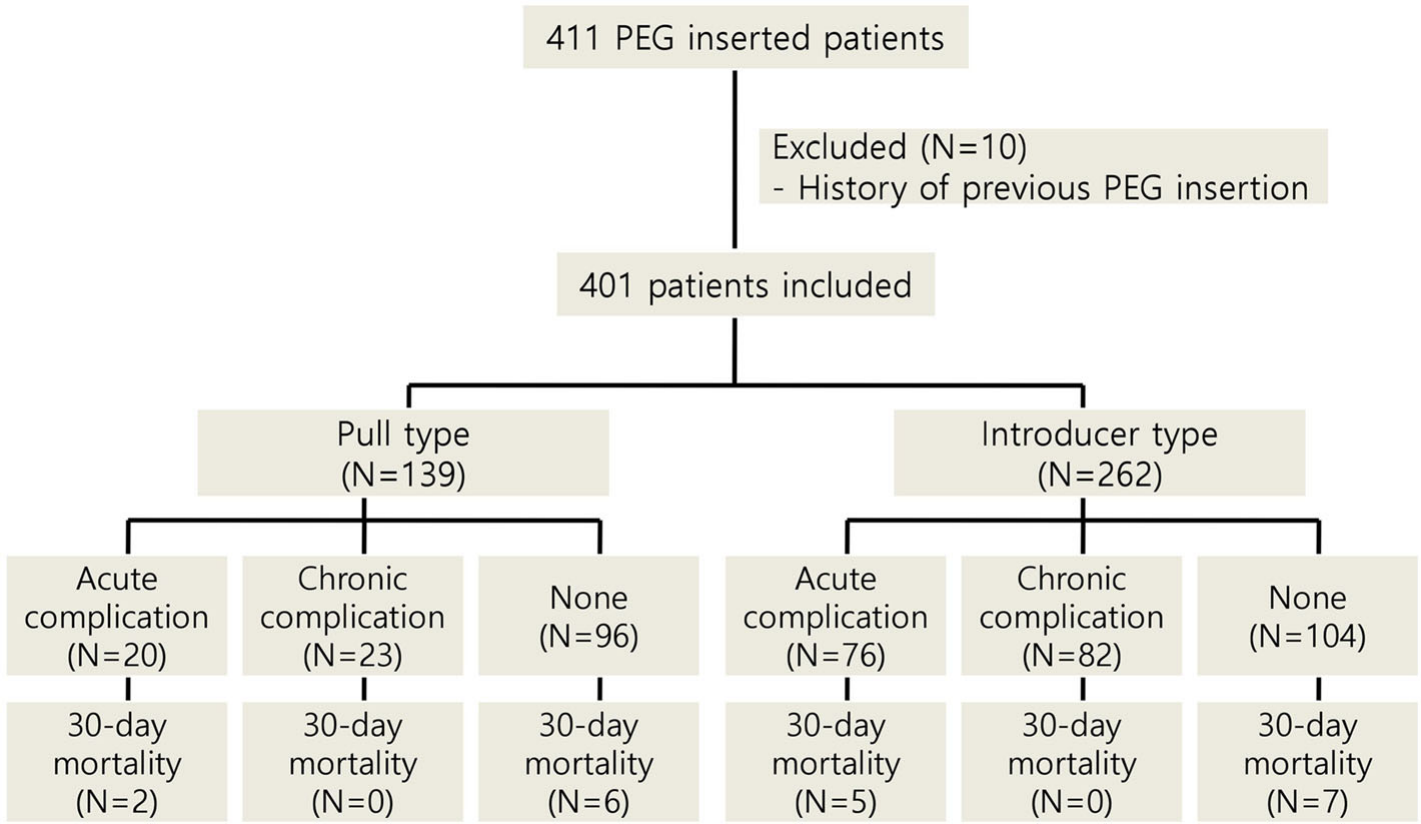
Study flow chart

A total of 411 patients who underwent PEG at the Asan Medical Center in Seoul, Korea, between January 2005 and December 2015 were included in this retrospective study. Medical records of all patients were retrospectively reviewed for investigating clinical characteristics of patients. Demographic data, including underlying disease, body mass index (BMI), indications for PEG, complications after PEG, comorbidities, and baseline laboratory values, were assessed. After exclusion of ten patients with a history of previous PEG insertion, 401 patients were included in the final analysis. A flow chart of the study population is shown in Fig 1. The study protocol was reviewed and approved by the institutional review board of the Asan Medical Center, Ulsan College of Medicine.

### PEG methods

**Fig. 2:**
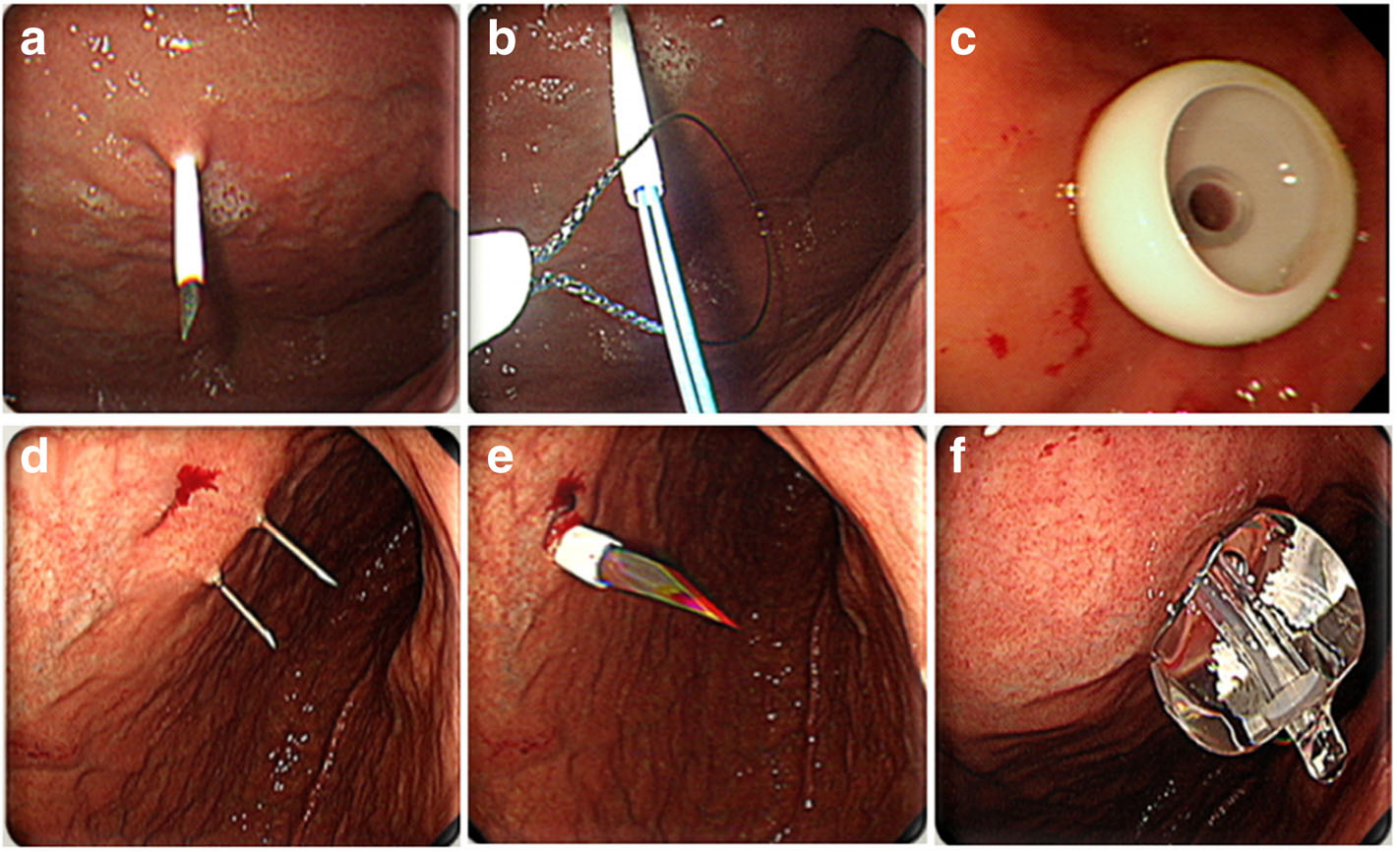
Percutaneous endoscopic gastrostomy (PEG) using the pull (**a–c**) or the introducer (**d–f**) technique. **a** A plastic sheath is inserted through the abdominal and gastric walls. **b** Snare grasping guidewire through the endoscopic channel. **c** PEG tube is placed by the pull technique using an internal bumper. **d** Double-lumen gastropexy needle in the stomach. **e** Penetration of the gastric wall using trocar and a peel-away sheath. **f** Fixation by inflation of the balloon with saline

PEG was performed by either the pull (i.e., Ponsky-Gauderer) technique using a 20- or 24-Fr PEG tube (US Endoscopy®, Mentor, OH, US), or the introducer (Russell) technique using a 15-Fr Cliny® PEG tube (Create Medic Co., Ltd., Yokohama, Japan) (Fig. 2). PEG insertion was performed by gastroenterologists in all patients. Among the 401 patients, the pull type technique was performed in 139 (34.7%) patients and the introducer type was administered in 262 (65.3%) patients.

At the Asan Medical Center, all patients underwent the pull-type PEG until November 2010; from December 2010 to October 2011, both techniques were used, but from November 2011, all but three patients received the introducer-type PEG.

PEG insertion by the pull technique was initiated with insertion of a transilluminated endoscope to determine the puncture site on the abdominal wall, with endoscopic monitoring of the anterior gastric wall. A guidewire was introduced through the puncture site and drawn out through the esophagus and oral cavity using biopsy forceps. The PEG tube was then connected to the guidewire and reinserted through the oral cavity, pulled down, and extracted through the puncture site on the abdominal wall. An immediate endoscopic examination was performed to confirm the appropriate placement of the PEG tube.

PEG insertion by the introducer technique was also initiated using a transilluminated endoscope; however, this technique used a gastropexy device and trocar to penetrate the abdominal wall and did not require the PEG tube to be pulled through the oral cavity. Following puncture of the gastric wall, the PEG tube was directly inserted through the abdominal wall and fixed by filling the tube balloon with 5 cc of saline.

Three hundred seventy seven (94.0%) patients received prophylactic antibiotic 30 min before the procedure. Most of the prophylactic antibiotic regimen was 2 g of cefazolin. However, patients who were already receiving antibiotic treatment were not required to change the antibiotic regimen unless the current regimen was not effective against gram-positive cocci. Before the procedure, all patients were sedated with intravenous midazolam (0.05 mg/kg) and pethidine (25 or 50 mg).

### Definitions

Post-procedural complications were classified into two groups according to the time of occurrence. Acute complications were defined as those developing ≤7 days after PEG, whereas chronic complications included those that occurred > 7 days after the procedure. Bleeding, pneumoperitoneum, aspiration pneumonia, ileus, wound infection, and Mallory-Weiss tear were classified as acute complications. Chronic complications included tube leakage, tube obstruction, spontaneous tube removal, wound infection, buried bumper syndrome, and recurrent aspiration pneumonia.

Bleeding was defined as an event requiring intervention (such as hemoclipping, embolization, or epinephrine injection) to control bleeding and follow-up endoscopic examination to check lesion healing. In addition, bleeding complications included mucosal tear bleeding and bleeding requiring compression during and after the procedure. Aspiration pneumonia was defined as the presence of a newly developed symptom (such as cough, purulent sputum, and fever) with indicative chest X-ray image change after the PEG procedure. Ileus included symptomatic and non-symptomatic cases with indicative X-ray or computed tomography (CT) image of ileus and decreased bowel sounds.

### Post-PEG management

On the day of the PEG procedure, all patients were kept NPO with natural tube drainage. On post-PEG day 1100 mL of clear water was flushed via the gastrostomy tube in patients whose abdomen was soft and in those who showed no abdominal discomfort. All patients underwent routine abdominal and chest X-ray on the same day as the procedure or the day after the procedure to check the position of the tube, as well as to detect any complications, such as pneumoperitoneum, aspiration pneumonia, and ileus. If any complications were detected, further imaging study or follow-up X-ray was performed. Patients who tolerated the water flush without abdominal pain or vomiting and had no evidence of leakage at the site of PEG were able to receive tube feeding with 100–200 mL of fluid.

### Statistical analysis

Descriptive data are expressed as mean ± standard deviation or median and interquartile range (IQR). Statistical analysis was performed using SPSS version 21.0. Fisher’s exact test and the chi-square test were used to determine differences between categorical variables. Logistic regression analysis was used to evaluate predictors of complications and 30-day mortality. A *p* value < 0.05 was considered to be statistically significant for all analyses.

## Results

### Baseline characteristics

Age, gender, BMI, indications for PEG, comorbidities, and baseline laboratory values for the study subjects are summarized in Table 1. The median age was 68 (IQR, 57–77) years, and median BMI was 19.5 (IQR, 17.2–21.7) kg/m^2^. There were 262 (65.3%) male patients in the study group. Indications for PEG were classified into three categories: patients undergoing PEG for neurologic disease (*n* = 240, 59.9%) included patients with stroke (*n* = 85), Parkinson’s disease (*n* = 61), neuromuscular disease (*n* = 53), dementia (*n* = 24), and hypoxic brain damage (*n* = 17); patients who underwent PEG for malignancy (*n* = 70, 17.4%) included patients with esophageal and head and neck cancer (*n* = 21 and 49, respectively). In addition, there were 14 patients with lung cancer, three with stomach cancer, and one with hematologic cancer, colon cancer, pancreatic cancer, spine tumor, and breast cancer, respectively.

**Table 1:** Baseline patient characteristics

	Total (*n* = 401)	Pull type (*n* = 139)	Introducer type (*n* = 262)	*p* value
Clinical characteristics				
Age (median, IQR)	68 (57–77)	67 (55–76)	70 (57–77)	0.404
Gender, male (%)	262 (65.3)	82 (59)	180 (68.7)	0.052
BMI (median, IQR)	19.5 (17.2–21.7)	20.1 (17.3–23.0)	19.3 (17.1–21.2)	0.017
Indications for PEG, n (%)				
Neurologic disease	240 (59.8)	85 (61.2)	155 (59.2)	0.645
Malignancy	70 (17.4)	29 (20.8)	41 (15.7)	0.190
Other	91 (22.7)	25 (18.0)	66 (25.2)	0.086
Comorbidities (%)	212 (52.9)	77 (55.4)	135 (51.5)	0.460
Antibiotic prophylaxis, n (%)	377 (94.0)	124 (89.2)	253 (96.6)	0.003
Concurrent medication, n (%)				
Aspirin	63 (15.7)	26 (18.7)	37 (14.1)	0.230
Clopidogrel	26 (6.5)	10 (7.2)	16 (6.1)	0.674
Warfarin	12 (3.0)	4 (2.9)	8 (3.1)	0.922
Others	23 (5.7)	8 (5.8)	15 (5.7)	0.990
Laboratory values				
WBC/μL	8243.0 ± 3580.2	8083.4 ± 3809.8	8324.6 ± 3461.6	0.528
Hemoglobin, g/dL	11.2 ± 2.0	11.2 ± 1.9	11.2 ± 2.0	0.987
Platelets, × 10^3^/μL	266.1 ± 119.1	276.5 ± 137.7	260.8 ± 108.3	0.216
Albumin, g/dL	3.0 ± 0.7	3.0 ± 0.6	3.0 ± 0.7	0.925
Prothrombin time, INR	1.06 ± 0.27	1.03 ± 0.27	1.07 ± 0.27	0.826
CRP, mg/dL	3.0 ± 3.9	3.4 ± 4.4	2.9 ± 3.7	0.283

In the overall study group, 186 (46.4%) patients had comorbidities, including hypertension (*n* = 173), diabetes mellitus (*n* = 90), and chronic kidney disease (CKD, *n* = 21). Antibiotic prophylaxis was used in 377 (94.0%) patients. Analysis of medication history for antiplatelet agents and anticoagulants revealed that 101 (25.2%) patients were treated with aspirin, clopidogrel, or warfarin; 12 patients (3.0%) were treated with dual aspirin/clopidogrel medication; and 11 patients had discontinued aspirin and clopidogrel at least 5 days before the procedure. None of the cases of bleeding were reported in patients treated with dual antiplatelet agents. Among 51 patients who were taking aspirin without clopidogrel, 32 patients had discontinued aspirin at least 7 days before the PEG procedure and 19 patients discontinued treatment less than 7 days before the procedure. However, bleeding was reported in only one patient who discontinued aspirin less than 7 days before the procedure. In 14 patients treated with clopidogrel without aspirin, 11 patients had discontinued medication at least 5 days before the procedure. Although three patients discontinued treatment less than 5 days before the procedure, no bleeding events were reported.

### Clinical outcomes

The median follow-up time was 354 (IQR, 79–787) days. Post-PEG complications are summarized in Table 2. With the exception of ileus and chronic tube obstruction, there were no significant differences in the incidence of acute or chronic complications between the pull- and introducer-type PEG groups (Table 2).

**Table 2:** Acute and chronic complications of percutaneous endoscopic gastrostomy according to the insertion type

Variable	Pull type (*n* = 139)	Introducer type (*n* = 262)	*p* value
Follow-up, days	524 (100–1178)	269 (64–686)	0.001
Acute complications, n (%)			
Bleeding	5 (3.6)	18 (6.9)	0.180
Pneumoperitoneum	9 (6.5)	29 (11.1)	0.135
Aspiration pneumonia	1 (0.7)	2 (0.8)	1.000
Ileus	4 (2.9)	22 (8.4)	0.033
Wound infection	1 (0.7)	1 (0.4)	1.000
Mallory-Weiss tear	0	4 (1.5)	0.303
Chronic complications, n (%)			
Wound infection	6 (4.3)	12 (4.6)	0.903
Leakage	7 (5.0)	22 (8.4)	0.216
Tube obstruction	5 (3.6)	37 (14.1)	0.001
Spontaneous removal	2 (1.4)	8 (3.1)	0.505
Buried bumper syndrome	1 (0.7)	1 (0.4)	1.000
Aspiration pneumonia	2 (1.4)	2 (0.8)	0.612
30-day mortality, n (%)	8 (5.8)	12 (4.6)	0.607

Acute post-PEG bleeding was observed in 23 (5.7%) patients. Although no additional intervention was required in the majority of cases, two patients received epinephrine injection, three required hemoclipping, and one underwent embolization to manage bleeding.

Pneumoperitoneum developed in 38 (9.5%) patients within the first week of PEG. Of eight patients with symptomatic pneumoperitoneum who presented with the signs of peritoneal irritation as well as imaging abnormalities on chest X-ray or CT, none required surgical intervention; all patients showed improvement with conservative management.

Ileus was observed in 26 (6.5%) patients, but none required surgical management. Wound infection was detected in 18 (4.5%) patients, among which three patients required not only antibiotic treatment but also removal of the PEG tube. Leakage was present in 29 (7.2%) patients, 26 of whom underwent PEG tube change.

### Risk factors for complications

Multivariate analysis revealed that underlying malignancy was a predictor of acute complications (odds ratio [OR], 2.205; 95% confidence interval [CI], 1.197–4.061; *p* = 0.011) and that age ≥ 70 years (OR, 1.022; 95% CI, 1.003–1.042; *p* = 0.021) and diabetes mellitus (OR, 1.877; 95% CI, 1.058–3.331; *p* = 0.031) were predictors of chronic complications (Tables 3 and 4).

**Table 3:** Univariate and multivariate logistic regression analysis of predictors of acute complications associated with percutaneous endoscopic gastrostomy

Predictor	Univariate analysis		Multivariate analysis	
	OR	*p* value	OR (95% CI)	*p* value
Clinical characteristic				
Age ≥ 70 years	1.129	0.615		
BMI < 19 kg/m^2^	0.955	0.196		
Diabetes mellitus	0.590	0.099		
Hypertension	1.002	0.993		
Chronic kidney disease	0.578	0.389		
Aspirin	0.546	0.114		
Clopidogrel	1.072	0.885		
Warfarin	0.705	0.655		
Indication for PEG				
Neurologic disease				
Stroke	0.580	0.098		
Other neurologic diseases	0.920	0.744		
Malignancy	2.003	0.017	2.205 (1.197–4.061)	0.011
Laboratory values				
WBC ≥ 12,000/μL	0.694	0.347		
Platelet count < 100,000/μL	1.163	0.798		
Prothrombin time ≥ 1.3	1.158	0.783		
Albumin < 3.0 g/dL	1.333	0.243		
Hemoglobin < 11 g/dL	1.390	0.175	1.029 (1.000–1.059)	0.054
CRP ≥ 5 mg/dL	1.240	0.484		
Procedure time	1.031	0.037		
Diameter of PEG tube	0.843	0.412

**Table 4:** Univariate and multivariate logistic regression analysis of the predictors of chronic complications associated with percutaneous endoscopic gastrostomy

Predictor	Univariate analysis		Multivariate analysis	
	OR (95% CI)	*p* value	OR (95% CI)	*p* value
Clinical characteristic				
Age ≥ 70 years	2.366	0.001	1.974 (1.186–3.285)	0.009
BMI < 19 kg/m^2^	1.161	0.542		
Diabetes mellitus	1.851	0.022	1.731 (1.007–2.977)	0.047
Hypertension	1.857	0.011		
Chronic kidney disease	0.570	0.376		
Aspirin	1.985	0.022		
Clopidogrel	1.316	0.550		
Warfarin	1.174	0.812		
Indication for PEG				
Neurologic disease				
Stroke	1.012	0.968		
Other neurologic diseases	1.580	0.063		
Malignancy	0.337	0.009	0.473 (0.199–1.082)	0.075
Laboratory values				
WBC ≥ 12,000/μL	0.419	0.054	0.434 (0.176–1.070)	0.070
Platelet count < 100,000/μL	0.226	0.152		
Prothrombin time ≥ 1.3	0.869	0.806		
Albumin < 3.0 g/dL	1.048	0.849		
Hemoglobin < 11 g/dL	0.837	0.470		
CRP ≥ 5 mg/dL	0.643	0.154		
Procedure time	0.611	0.153		
Diameter of PEG tube				
15-Fr		0.031		
20-Fr	0.143 (0.034–0.607)	0.008		
24-Fr	0.976 (0.434–2.194)	0.952		

Subgroup analysis was conducted to compare the malignancy group and neurologic disease groups. No significant differences in procedure time, PEG type, and acute complication rate were seen between the two groups, with the exception of the incidence of pneumoperitoneum (*p* = 0.016; Table 5). However, all pneumoperitoneum events were benign and improved with conservative care.

**Table 5:** Subgroup analysis between malignancy group and neurologic disease group

Variable	Malignancy	Neurologic disease	*p* value
Acute complication, n (%)			
Bleeding	3 (4.3)	12 (5.0)	1.000
Pneumoperitoneum	12 (17.1)	18 (7.5)	0.016
Aspiration pneumonia	2 (2.9)	1 (0.4)	0.129
Ileus	5 (7.1)	15 (6.3)	0.784
Wound infection	0	1 (0.4)	1.000
Mallory-Weiss tear	0	3 (1.3)	1.000
Pull-type PEG, n (%)	29 (41.4)	85 (35.4)	0.359
Procedure time, minutes	14.5 (10.3–18.0)	16.00 (11.0–20.0)	0.128

### Prognosis

The 30-day mortality rate was 5.0%, and 20 patients died within 30 days of PEG insertion. On multivariate analysis, platelet count < 100,000/μL (OR, 14.294; 95% CI, 3.358–60.851; *p* = 0.000), C-reactive protein (CRP) ≥ 5 mg/dL (OR, 3.101; 95% CI, 1.021–9.414; *p* = 0.046), and the other neurologic disease group were found to be significant factors associated with 30-day mortality (OR, 0.125; 95% CI, 0.016–0.994; *p* = 0.049) (Table 6). The other neurologic disease group (including dementia, Parkinson’s disease, neuromuscular disease, and hypoxic brain damage) was a significant factor that showed a lower incidence of 30-day mortality than stroke and malignancy groups.

**Table 6:** Univariate and multivariate analysis for determining factors associated with 30-day mortality after percutaneous endoscopic gastrostomy

Predictor	Univariate		Multivariate	
	OR (95% CI)	*p* value	OR (95% CI)	*p* value
Clinical characteristic				
Age ≥ 70 years	0.721	0.485		
BMI < 19 kg/m^2^	0.775	0.607		
Diabetes mellitus	0.596	0.418		
Hypertension	0.549	0.230		
Chronic kidney disease	5.353	0.006	3.990 (0.993–17.072)	0.062
Aspirin	0.271	0.207		
Clopidogrel	0.749	0.783		
Warfarin	0.000	0.999		
Indication for PEG				
Neurologic disease				
Stroke	0.926	0.893		
Other neurologic diseases	0.094	0.022	0.125 (0.016–0.994)	0.049
Malignancy	1.193	0.759		
Laboratory values				
WBC ≥ 12,000/μL	1.167	0.811		
Platelet count < 100,000/μL	21.778	0.000	14.294 (3.358–60.851)	0.000
Prothrombin time ≥ 1.3	2.170	0.324		
Albumin < 3.0 g/dL	2.227	0.113		
Hemoglobin < 11 g/dL	3.950	0.009		
CRP ≥ 5 mg/dL	2.994	0.032	3.101 (1.021–9.414)	0.046
Procedure time	0.991	0.751		
Diameter of PEG tube		0.813		

Among the 20 patients who died within 30 days of PEG, the cause of death was identified in 12 patients. The most common cause of death was pneumonia (*n* = 7). Mortality in two patients was considered to be associated with the PEG insertion procedure. One patient was admitted to the hospital to undergo chemotherapy for breast cancer with brain metastasis and leptomeningeal seeding; the patient presented with radiologic changes indicative of aspiration pneumonia and desaturation the day after PEG insertion and died 12 days after PEG as a result of aggravation of pneumonia. The other patient suffered from liver cirrhosis due to hepatitis B and C combined autoimmune hepatitis and was admitted to the hospital because of hepatic encephalopathy. The patient presented with fever on the day of PEG insertion and showed signs and symptoms of peritonitis with supporting diagnostic paracentesis result, which was normal the day before PEG. However, abdomen-pelvis CT showed no signs of peritonitis and the patient died 5 days after PEG as a result of uncontrolled infection. In addition, three patients presented with aggravation of previous pneumonia after PEG without evidence of a correlation with PEG; three patients developed aspiration pneumonia at least 10 days after PEG insertion. Other causes of mortality unrelated to the PEG procedure included peritonitis, uncontrolled bleeding, liver failure, kidney failure, and traumatic brain injury. The cause of death was unknown in eight patients.

## Discussion

PEG is a common procedure indicated for patients with normal gastrointestinal function who are expected to require prolonged enteral feeding. However, patients who require PEG usually have chronic underlying diseases and vulnerable general condition. Although there are currently no established standard criteria for patients requiring PEG, guidelines published by the American Gastroenterological Association recommend that PEG is performed only in patients who are expected to survive for more than 30 days after the procedure [10]. Despite continuing efforts to evaluate risk factors associated with PEG-related complications and mortality, several studies have reported different risk factors [8, 11–14]. In addition, although PEG has been shown to be a safer approach than radiological or surgical insertion, the complication rate with PEG is reported to be 13.2–42.9% [12, 15, 16]. Complications including bleeding, wound infection, tube blockage, tube leakage, aspiration pneumonia, perforation, and buried bumper syndrome are associated with PEG [17].

In the present study, the technique of tube insertion was not associated with the rate of acute or chronic complications, with the exception of tube obstruction and ileus. The rate of tube obstruction was significantly higher with introducer- than pull-type PEG (3.6% vs. 14.1%, *p* = 0.001). As the introducer-type PEG uses a thinner 15-Fr tube than the pull-type PEG (20- or 24-Fr tube), our finding suggests that the use of a thinner tube is associated with a higher obstruction rate. The European Society for Clinical Nutrition and Metabolism guidelines recommend that PEG tubes should be at least 15 Fr to minimize the risk of tube obstruction [1].

In addition, the rate of ileus was significantly higher in the introducer-type PEG group than the pull-type PEG group (2.9% vs. 8.4%, *p* = 0.033). Transient ileus rarely occurs after PEG, with a reported rate of 1–2% [18]. In the present study, all patients with ileus showed improvement after conservative management. Diabetes mellitus may contribute to an increase in gastroparesis or ileus, but no significant difference in the rate of diabetes mellitus was seen between the two groups (25.2% vs. 21.0%, *p* = 0.339). Conversely, analysis for underlying diseases revealed that the percentage of patients with neuromuscular disease was significantly higher in the introducer-type group (7.2% vs. 16.4%, *p* = 0.009). Neuromuscular diseases, such as amyotrophic lateral sclerosis, are associated with delayed gastric emptying and prolonged colonic transit time, which may contribute to the development of post-PEG ileus [19, 20].

In the present study, older age and diabetes mellitus were risk factors associated with chronic complications of PEG. Old age with debility and senility is a well-known factor affecting various medical conditions, and a previous study showed old age to be a risk factor for early mortality after PEG [11]. Because aging is associated with immune system imbalance, infection-related complications may increase in older patients [21, 22]. In the present study, infection-related chronic complications (including wound infection and recurrent pneumonia) comprised 21% of all chronic complications after PEG.

Diabetes mellitus is considered to be a significant risk factor associated with wound infection after invasive procedures and surgery. Furthermore, diabetes mellitus alters immunity by suppressing polymorphonuclear leukocyte function and cutaneous responses to antigen challenges [23]. In the present study, wound infection in the chronic period following PEG occurred in 18 (4.5%) patients. Given that PEG is an invasive procedure with penetration from the skin to the stomach, wound healing is crucial for the prevention of infection and tube leakage. In this study, significant increases in chronic complications associated with wound healing were seen in patients with diabetes mellitus underwent PEG. In addition, tube leakage was the second most common chronic complication, developing in 29 (7.2%) patients. Diabetes mellitus may also have contributed to the tube leakage observed in nine patients who presented with signs of inflammation, such as redness, pain, swelling, and pus-like discharge at the insertion site.

Several risk factors, such as elevated CRP level, low albumin level, older age, low BMI, and diabetes mellitus, have been reported to be associated with early mortality after PEG [8, 11–14]. In the present study, 30-day mortality was associated with high CRP levels, low platelet count, and neurologic diseases. In a prospective cohort study, Blomberg et al. reported that the combination of low albumin and high CRP was a predictor of 30-day mortality after PEG [14]. Additional studies reported elevated CRP level as an independent risk factor for 30-day mortality after PEG [13, 16].

In the current study, CRP which is an acute phase reactant was not significantly associated with acute complications. The most common acute complications after PEG were pneumoperitoneum, bleeding, and ileus, which are not associated with active inflammation or infection. Pneumoperitoneum following PEG occurs frequently and is usually a benign finding without peritoneal inflammation [24]. Also, in the present study, all of the patients with pneumoperitoneum improved with conservative management. Furthermore, infection-related complications (such as aspiration pneumonia and wound infection) were seen in only 0.7% and 0.5% of patients, respectively.

By contrast, CRP was a significant factor for 30-day mortality after PEG. CRP is a marker of inflammation and severity of the underlying disease and has been reported as a prognostic factor for various different disease entities, including cardiovascular disease, renal failure, ischemic stroke, chronic obstructive pulmonary disease, acute pancreatitis, and postoperative complications [25–30]. Considering the fact that, in 8 of the 20 patients who died within 30 days of PEG, death was associated with infection, pneumonia, or peritonitis, elevated CRP levels may be significantly associated with post-PEG prognosis.

In this study, stroke (21.2%), malignancy (17.5%), and Parkinson's disease (15.2%) were the most common underlying diseases. In stroke patients, CRP was reported as a predictor not only of early mortality but also long-term mortality [31, 32]. In addition, an association between baseline CRP and early death after diagnosis of any cancer has been reported [33]. Moreover, baseline CRP has also been reported as a predictor of mortality in patients with Parkinson’s disease [34]. In this study, patients had chronic underlying diseases, including stroke, cancer, and Parkinson’s disease, and CRP was a significant factor predicting early mortality, as seen in previous studies.

In addition, platelet count was also associated with early mortality after PEG in the present study. Multivariate analysis showed that low platelet count significantly increased the risk of 30-day mortality. Platelet is a known local host defense factor against endovascular infections, as demonstrated in an animal model of induced thrombocytopenia, which was associated with disease progression [35]. Also, platelet count was reported as an independent prognostic factor of mortality in patients with sepsis as well as in critically ill patients [36, 37].

The present study has several limitations. First, because of the retrospective design of the study, some data were missing from the medical records. In addition, the rate of complications and mortality was determined only by chart review. Therefore, the potential for incorrect assessment of complications and cause of death cannot be excluded. Secondly, the median follow-up period was relatively shorter than total research periods. As a tertiary hospital, we usually transfer patients to a local hospital or nursing hospital after an active phase of disease. In this study, the median number of tube changes after PEG was only 1.07. Third, the present study was conducted at a tertiary referral hospital where the disease severity is higher than that seen in general hospitals. Inevitable ethical problems and the challenges associated with designing large, randomized controlled studies mean that trials of gastrostomy are rare.

## Conclusions

In conclusion, this study of data collected over a long time frame shows that PEG is a relatively safe and feasible procedure. Patients in neurologic diseases group including dementia, Parkinson’s disease, neuromuscular disease, and hypoxic brain damage showed significantly lower early mortality rate than other diseases group (including stroke and malignancy). However, platelet count < 100,000/μL, or CRP ≥ 5 mg/dL were risk factors associated with early mortality after PEG. In addition, acute complications in patients with underlying malignancy, and chronic complications in patients aged ≥70 and those with diabetes mellitus should be considered during and after PEG.
